# Impact of COVID-19 and related measures on the professional life of school staff based in Switzerland: challenges, strategies, and benefits

**DOI:** 10.1093/eurpub/ckae196

**Published:** 2024-12-09

**Authors:** Marta Fadda, Guenda Bernegger, Kleona Bezani, Ilaria Falvo, L Suzanne Suggs, Maria Caiata-Zufferey

**Affiliations:** Institute of Public Health, Università della Svizzera italiana, Lugano, Switzerland; Institute of Family Medicine, Università della Svizzera italiana, Lugano, Switzerland; Department of Business Economics, Health and Social Care, University of Applied Sciences and Arts of Southern Switzerland, Manno, Switzerland; Institute of Public Health, Università della Svizzera italiana, Lugano, Switzerland; Institute of Public Health, Università della Svizzera italiana, Lugano, Switzerland; Institute of Communication and Public Policy, Università della Svizzera italiana, Lugano, Switzerland; Department of Business Economics, Health and Social Care, University of Applied Sciences and Arts of Southern Switzerland, Manno, Switzerland

## Abstract

To reduce the spread of COVID-19, schools closed and moved to remote learning in many countries and municipalities. At the time of this study, Switzerland maintained the position that schools should be prioritized to remain open. The state of knowledge regarding the challenges that school staff encountered during the pandemic was limited. The aim of this study was to qualitatively explore the impact of COVID-19 and related measures in Switzerland on the professional life of school staff in terms of challenges, strategies, and benefits. Data were collected between November 2021 and March 2022. We conducted interviews with 47 participants working in nurseries, kindergartens, primary, middle, special, and after-schools across Switzerland. The majority were women and part of the teaching staff. Most had received at least one dose of the COVID-19 vaccine. Participants reported various challenges such as isolation, loneliness, and ‘dryness’ of work, pedagogical alienation and deontological distress, privacy loss, and confusion regarding health measures. They also cited different strategies, namely resuming simple activities, finding the right degree of transgression, recreating normality with children, and continuous adaptation. Finally, they identified some benefits, i.e. improvements in education regarding hygiene standards, greater acceptance of illness and death as a normal part of life, recognition of the privilege of being able to attend school in-person, and valorization of cultural diversity. We highlight the importance of listening to staff’s concerns, enacted strategies, and perceived benefits and taking them into account when developing appropriate and effective public health and communication efforts.

## Introduction

Given the crucial role that school structure plays in the well-being of youth and the economy, teachers and school staff are a unique population in the effort to minimize societal disruptions and inequities resulting from outbreaks of infectious diseases and other crises affecting school staff and students. When schools are disrupted, education and other critical functions are compromised, disproportionately impacting vulnerable populations, including low-income families and students with special needs.

School closures and the transition to distance learning during the initial wave of the COVID-19 pandemic had significant negative impacts on income, mental health, and learning for families and youth, disproportionately impacting low-income families and those requiring essential support coordinated through the public-school system globally [1, 2]. At the time of this study, Switzerland maintained the position that schools should be prioritized to remain open. Schools remained open during the second and following waves of the pandemic. However, exposure events, compulsory use of masks, and quarantine guidelines continued to be disruptive for staff, students, and families alike.

A growing body of research indicates that school staff, particularly teachers, faced multiple stressors as they adapted to pandemic-related changes. Specifically, they faced an increased workload, being subjected to media scrutiny and debate, and adapting to new technologies and work methodologies [3, 4]. Furthermore, pandemic-related measures impacted the quality of interactions between teachers and students. Social distancing requirements prevented the physical closeness that is often required for effective teaching and mentoring [5]. Further, the COVID-19 pandemic resulted in significant disruptions to students’ learning and social-emotional development, which placed more pressures on teachers to provide additional support and resources to their students [6]. Thus, a major professional challenge many teachers faced was to guarantee continuity of high-quality education and support the socio-emotional needs of learners at the same time [7]. The pandemic also took a heavy toll on school staff’s mental health, well-being, and quality of life [8, 9]. School teachers experienced higher anxiety, poorer mental health, and higher levels of psychological distress, depression, and burnout compared to pre-pandemic evidence, with a greater impact on women [10–13]. Data also showed declining levels of enjoyment of work, support, and connection at work [4], a significant decrease in teachers’ quality of life, with a greater impact among women [14], and increased struggles to have a satisfactory work-family balance [11, 15]. These challenges inevitably affected the organizational commitment levels of teachers which significantly decreased after the implementation of COVID-19 pandemic measures. The impact of COVID-19 on school staff was not distributed evenly across the board. For example, teachers and principals in lower-income schools were more likely to face challenges related to technological resources and connectivity during the transition to remote learning, while also facing higher levels of stress and workload due to the increased needs of historically marginalized students in their communities [4, 6, 16]. Moreover, staff who belong to historically marginalized groups may have faced greater personal health risks, while also experiencing the compounding effects of systemic inequities in education and healthcare systems [17].

While research has begun to shed light on the professional challenges experienced by school staff, less is known about how they responded to these challenges and what opportunities the pandemic may have also introduced. This study aimed to qualitatively explore the impact of COVID-19 and related measures on the professional life of school staff based in Switzerland in terms of professional challenges, respective strategies, and professional benefits.

## Methods

Drawing from the comprehensive sociological tradition of Max Weber [18], we conducted a qualitative study [19] using individual interviews with a sample of staff employed in public and private nurseries, kindergarten, primary, middle, and after-schools in both urban and rural areas in the three linguistic regions of Switzerland. Capturing an authentic and comprehensive representation of experiences was the primary objective of this qualitative study, instead of offering a comparative representation of them [20]. For this reason, we diversified the sample according to the linguistic regions where participants were based. The interview aimed to understand participant’s professional challenges in relation to the pandemic and its measures, their strategies to address them, and the possible benefits stemming from managing such challenges. An in-depth, semi-structured approach was followed, and an interview grid was developed on the basis of interview grids successfully employed by the project team in previous projects and the scientific literature on the impact of COVID-19 (see Supplementary file). Four trained qualitative interviewers, with experience in remote qualitative research [21], conducted the interviews in each participant’s preferred language (German, French, or Italian). The data collection and the analysis proceeded simultaneously to allow emerging themes to inform subsequent interviews [22]. The project was authorized by the Ethics Committee of the Canton of Ticino (ID 2021-01955) on 26 October 2021.

We included individuals filling a quantitative online survey on COVID-19 professional challenges and COVID-19 vaccine hesitancy and who consented to participate in a follow-up interview (see Supplementary file). Additionally, snowball sampling methods were employed using personal contacts, particularly in the German- and French-speaking regions of Switzerland. Interviews were conducted over the phone, after oral informed consent, between November 2021 and March 2022 and subsequently transcribed verbatim.

We employed thematic content analysis with a mainly inductive approach to analyse the data [23], enriched with abductive and deductive reasoning [24] (see Supplementary file). We follow the consolidated criteria for reporting qualitative research [25].

## Results

### Sociodemographic characteristics of participants

The final sample included 47 participants, of which 37 were women. Nineteen were based in the Italian-speaking Canton of Ticino, 15 in a French-speaking Canton, and 14 in a German-speaking Canton (see Table 1). The mean age was 42.3 in Ticino, 41.9 in French-speaking, and 54.1 in German-speaking Switzerland. Twelve worked in a kindergarten, 28 in a primary school, 3 in a middle school, and 2 in a special needs school. Of the entire sample, 36 were teaching staff, 7 were non-teaching staff, and 4 worked in a managerial position. In terms of location, 26 were based in an urban area and 18 in a rural one. Thirty-six of the 47 were vaccinated against COVID-19 with at least one dose, while 11 were not vaccinated. Participants listed four main professional challenges, four professional strategies, and four professional benefits. Each theme is elucidated in the following paragraphs with relevant quotations from the participants (see Table 2).

**Table 1.** Sociodemographic characteristics of participants

*N* = 47	Italian-speaking *linguistic region* (*n* = 19)	French-speaking *linguistic region* (*n* = 15)	German-speaking *linguistic region* (*n* = 14)	Total
Female	15	11	9	37
Mean age	42.3	41.9	48.2	–
Type of school				
Kindergarten	9	1	2	12
Primary	10	12	6	28
Middle	–	1	3	4
Special	–	–	2	2
Role				
Teaching staff	14	13	10	37
Non-teaching staff	3	2	2	7
Managerial	2	–	2	4
Area				
Urban	10	4	9	23
Rural	9	8	5	22

**Table 2.** Illustrative quotes from participants

Themes and subthemes	Quote #	Supporting quotes
Professional challenges		
Isolation, loneliness, and ‘dryness’ of work		
	#1	*All the nice things are gone. (female, French-speaking linguistic region)*
	#2	*It was no longer possible to organize mixed activities between classes, there were no more events. We used to celebrate the beginning of the vacations the day before the vacations started. [..] We saw each other much less, there was less exchange. (female, German-speaking linguistic region)*
	#3	*We had to divide the courtyard into sectors so that the different classes did not mix during break. In some cases, we staggered the breaks. (female, German-speaking linguistic region)*
	#4	*As a teacher, it really feels strange to arrive in the classroom and see children wearing masks, with this thing over their mouths. For adults it makes sense, and you just adjust to it. But seeing children like that makes me very sad. I understand the reason and that’s fine. But being there all day and not seeing their expressions and their faces, if they have a sad face… This does not give me much satisfaction.* *(female, Italian-speaking linguistic region)*
Pedagogical alienation		
	#5	*It is like locking young people who are in the puberty stage or after puberty, [the period when] they fall in love. They are those who need to socialize, this is important for the peer group [group of belonging], like the days when I was studying in college. (male, German-speaking linguistic region)*
	#6	*They have become wild. For example, they have lost the habit of shaking hands when they meet other people. (female, German-speaking linguistic region)*
	#7	*For me it was very hard, because it was unimaginable to me to welcome new students with a mask. (female, French-speaking linguistic region)*
	#8	*I remember that in the spring of last year, in 2021, it happened several times that entire classes had to be quarantined. Almost every kindergarten or primary school class was affected: If there were more than two children per class who tested positive, the whole class had to go into quarantine. […] The classes are… Normally one third of the children are missing. (male, German-speaking linguistic region)*
	#9	*Actually, we have been teachers, psychologists, administrators, nurses. We have gone far beyond the teaching mandate. We really lost the basic job. (female, French-speaking linguistic region)*
	#10	*There were tense moments, for example, when we were asked to report parents returning from abroad who had not been tested in the red zone. That was something I didn't really get into. (male, French-speaking linguistic region)*
	#11	*It is very difficult to teach with the mask on because of the [difficulties in] understanding the language. Particularly in the field of language teaching, I find it very difficult. […] So we didn't have any big problems, but the lessons became a bit more difficult, definitely more difficult. (female, German-speaking linguistic region)*
Privacy loss		
	#12	*We as teacher had to fill a form to state if we were vaccinated or not. As far as I know, all teachers were vaccinated. But among the teachers for students with special needs, there was one who did not get vaccinated. (female, Italian-speaking region)*
	#13	*I think I am the only one who is not vaccinated. They all take their mask off when we are in the garden. I don’t. (female, Italian-speaking region)*
Confusion regarding health measures		
	#14	*The other difficulty is that the rules change all the time: What can be done, what cannot… (female, German-speaking linguistic region)*
	#15	*There was some confusion because the newspapers were mentioning [the introduction of new measures in educational settings], while we didn't know anything official yet. It was really bad and confusing. (female, Italian-speaking linguistic region)*
Professional strategies		
Resuming simple activities		
	#16	*We spent much more time outside playing with them. (female, German-speaking linguistic region)*
	#17	*We adapted, we can say we adapted. What was possible to do, we did it. What wasn't possible to do or what didn’t seem “human” to us, we didn't do it. (female, French-speaking linguistic region)*
	#18	*School outings were not allowed, you could not be out and about all day, but it was possible to spend two hours in the forest. In the end, we adapted. We were very grateful that we could do what we could do, despite everything. (female, German-speaking linguistic region)*
	#19	*We had time and we could role-play or do certain things that we usually never have time for. (female, German-speaking linguistic region)*
Finding the right degree of transgression		
	#20	*I think I will do the same as last year, i.e., mask all day and vaccination. When there are reading times, I will keep my distance and remove the mask to give the children a chance to read lips and emotions. For the rest, mask, disinfectant, and distances, and, where possible, I will offer the child the possibility of human contact. (female, Italian-speaking region)*
	#21	*I think I do what I'm asked to do within what is reasonable. I wash my hands, I wear the mask… But it's true that, for example, I always read a story to my pupils with the mask off, even when we weren't allowed to take the mask off. I think it's not possible [to do read it with the mask on]. I can't read a story to my pupils while they can't see the expression on my face. I can't say good morning to them in the morning with my mask on. Just for a few minutes a day, they have to see my face. Clearly, I could have been reprimanded. (female, French-speaking linguistic region)*
Recreating normality with children		
	#22	*I insist on hand washing, because I tell them that whether it's Corona or something else, germs are passed around all the time when we live together in a classroom. (female, French-speaking linguistic region)*
	#23	*We played games on the expression of the eyes. Through these games, children learn to tell from my eyes if they have done something wrong. They learn to recognise my angry expression from the eyes. Or if we laugh, they learn to read it from the eyes. (female, Italian-speaking linguistic region)*
Continuous adaptation		
	#24	*In particular, for the children with whom we have to work on the motorial skills of the mouth, we bought transparent masks, we installed transparent panels. […] It is amazing how the children adapted immediately. Really. They adopted things as they were. As far as language is concerned, I don't think they have made no progress. At the beginning, we were very critical regarding masks. We thought it wouldn't go well, especially with the little ones. But from my point of view everything worked incredibly well. (female, German-speaking linguistic region)*
	#25	*With the parents, the connection was more difficult to maintain, since we had very little time to discuss about the day, to raise sensitive issues: the opportunities to meet them were reduced, which slowed us down quite a bit and we had to find strategies to stay in communication with the families. (male, French-speaking linguistic region)*
	#26	*We had to be creative. We were no longer in our comfort zone, we had to adapt. (male, French-speaking linguistic region)*
	#27	*The regimes were so tight that you had a hard time adapting overnight. (female, German-speaking linguistic region)*
Secondary benefits		
Improvements in education regarding hygiene standards		
	#28	*If we can avoid putting our hands in our noses and touching everything, that's good. So if we touch our nose, we'll wash our hands (female, French-speaking linguistic region)*
	#29	*And there was also something that we had never seen before: It was the attention to wash the hands regularly… So, there really was… We see that this attention was reinforced.* (*male, French-speaking linguistic region)*
Greater acceptance of illness and death as a normal part of life		
	#30	*We take care of so many things that in the end we don't accept this death. […] We want to control everything, there isn't really any room. (female, French-speaking linguistic region)*
	#31	*The children have also heard such negative things that I thought I should show them that we will have to live with this, that we will not be able to eradicate this disease which is likely to come back frequently, like the flu, and to show the children that we can live with it serenely. (female, French-speaking linguistic region)*
Recognition of the privilege of being able to attend school in-person		
	#32	*The children were very happy to be able to go back to school. […] The joy of going to school is greater since the pandemic. The gratitude of being able to go to school [is higher]. (female, German-speaking linguistic region)*
	#33	*The school was re-evaluated. When the school was able to start again we got a lot of positive comments, “How nice that you are here again” and “We are happy” and… They realized that it is not always easy to work and teach their children, they also understood certain difficulties. For example, you have to repeat things many times, children don't learn so fast. They focused on making their children learn and that is also an experience, a positive experience. (female, German-speaking linguistic region)*
	#34	*They were all very happy to be able to go back to school after the lockdown. […] They were happy because they said: “We get bored at home.” (female, German-speaking linguistic region)*
Valorization of cultural diversity		
	#35	*And the Japanese said, “What? Switzerland is a developing country, in Japan there has been a mask requirement for a long time already, as well as restrictions. It's finally coming to Switzerland too!” (female, German-speaking linguistic region)*

### Professional challenges

Participants listed four challenges they encountered in their professional life as a consequence of COVID-19 and the measures to contain it.

#### Isolation, loneliness, and ‘dryness’ of work

Teachers reported a feeling of isolation with their class (#1). Some also stressed a lack of professional gratification following the implementation of protective measures (#2). These were due to a sharp reduction in complementary activities to school (field trips, visits, tandem work with other schools or classes, etc), separation of classes (#3), and lack of moments to eat together. The use of facial masks, which partially conceal the manifestation of affective responses, was mentioned by several participants as a potential contributing factor not solely to comprehension challenges, but also to emotional desensitization and diminished contentment, which we caterogized as ‘dryness’. (#4).

#### Pedagogical alienation and deontological distress

According to many participants, the public health management of the pandemic was ontologically at odds with the pedagogical mission of the school. This triggered deontological distress among a considerable number of teachers, yielding a sensation that we defined pedagogical alienation, whereby individuals find themselves unable to effectively carry out their duties while prioritizing pedagogical objectives. Some participants showed concern for young people (#5), stressing the negative consequences of the measures on their pupils (#6). Participants identified multiple reasons for this:

- The reduction of the conditions necessary for an effective pedagogical relationship: It was detrimental to have to wear a mask or maintain physical distance with younger children (#7);
- The waning of the collective dimension as a resource: Contagions and constant quarantines prevented the class from advancing collectively (#8); there was no longer the feeling of being a group (and this was an issue because participants noted that school is a collective activity);
- the internal conflict within one’s role: other social and health mandates took over the educational mandate (#9). Some participants reported discomfort over the enforced embracement of a control function (#10).
- The difficulty of responding to individual needs, particularly of those who are less linguistically comfortable. Some participants noted that included in the educational mandate is the role of promoting the integration of students and, in particular, children with speaking difficulties or who are not fluent in the language spoken by the rest of the class (#11).

#### Privacy loss

Many participants felt exposed in their personal choices in their workplace, especially regarding vaccination. There were times when the unvaccinated staff were required to wear a mask while the vaccinated were not, and this clearly exposed the participant’s personal choice, possibly also against their will (#12, #13).

#### Confusion regarding health measures

The constant changes and the multiple pieces of information regularly and suddenly reaching schools from different sources and including different levels of information, were perceived as confusing and created disorientation (#14, #15). Confusion came not only from the constant changes and the many levels of information, but also from knowing other schools or institutions with other approaches.

### Professional strategies

To cope with these challenges, participants developed four main professional strategies in order to continue to carry out their work in the best possible way.

#### Resuming simple activities

Some participants mentioned that they went back to doing simpler activities with the students that they had stopped engaging with (#16, #17, #18, #19). Some also reported resuming activities that they considered ‘essential’ and that had transformed into more complex activities recently.

#### Finding the right degree of transgression

Several participants reported that a certain level of transgression was necessary for them to perform their work. By transgression, they meant that breaking or bending certain rules or norms was sometimes required to achieve their teaching goals, help pupils and students complete their tasks effectively, ensure that the collective dimension was not lost (#20), and to not completely deprive children of facial expressiveness. For example, some decided to take off their mask when they were telling stories to younger children (#21).

#### Recreating normality with children

A number of participants attempted to re-establish a sense of normality in their lives by adopting the mindset that they were no longer in a state of exception but rather, in a new normal. As such, they recognized the need to integrate the health measures mandated by public health officials into their daily routines on a long-term basis. For instance, washing hands was viewed as a regular, habitual gesture that should be performed at all times, rather than solely during this period. Participants sought to transform these health measures from temporary precautions to enduring educational practices that could help promote a healthier and safer environment in the long run (#22, #23).

#### Continuous adaptation

Participants reported having to continuously and creatively find solutions to the challenges that arose. This required them to adapt the format of their classes, find compromises, and make changes to their teaching methods to ensure that students could continue to learn in a safe and effective manner. For example, teachers had to modify their lessons to allow for social distancing, reconfigure their classroom setups, and change the ways in which they interacted with students. Even where wearing the mask created greater disadvantages for learning, as in the case of speech therapy, suitable solutions were found that did not reduce the quality of work (#24). Participants reported they no longer organized school trips outside Switzerland but only within the country, and visited museums as individual classes rather than in school wide groups. The participants also emphasized the importance of maintaining the educational role of schools and finding ways to preserve the enjoyable aspects of school life. The ways of relating with families was also revisited to ensure quality and continuity despite the new constraints (#25). They recognized that social connections were a crucial component of the school experience, and sought to find ways to maintain these connections despite the challenges posed by the pandemic (#16). However, some participants noted that this was not always possible (#26, #27).

### Professional benefits

Alongside the distress and fatigue involved in the adjustment process, participants reported some benefits. These were partly the result of their effort and ability to turn necessity into a virtue and to transform some of the limitations introduced by the pandemic into opportunities.

#### Improvements in education regarding hygiene standards

Participants saw the implementation of COVID-19 measures as an opportunity to improve educational efforts on hand hygiene for the prevention of diseases other than COVID-19 (#28, #29).

#### Greater acceptance of illness and death as a normal part of life

Participants reported that the pandemic led to an increase in the acceptance of illness and death as two normal elements of life. One participant noted that the difficulty to accept illness and death is due to our desire, so frequent in Western societies, to have control of everything. The pandemic questioned this state of mind (#30, #31), allowing this issue to also be addressed in the school context.

#### Recognition of the privilege of being able to attend school in-person

The very value of schooling was re-evaluated. The return to in-person teaching after the first wave was welcomed with strong gratitude. The transition from remote to in-person learning allowed for a recognition of the beauty of physically attending school (#32, #33, #34).

#### Valorization of cultural diversity

Teachers highlighted differences in perspectives, cultures, and practices, for which the pandemic had provided an opportunity for appreciation. In classrooms with a high presence of children from various backgrounds, teachers valued good practices that had been already in use in other countries, such as the use of masks in Japan (#35).

## Discussion

This study aimed at understanding the impact of COVID-19 and related health measures on the professional life of school staff employed in Switzerland. We found that COVID-19 health measures introduced profound challenges on participants’ work. We recognized that participants employed a variety of professional strategies to manage such challenges, and identified professional benefits deriving from efforts to manage such challenges.

COVID-19 health measures had profound repercussions on participants’ professional lives, introducing challenges that caused distress and disorientation. Some of these challenges are not merely professional, but also have a profound moral character. The literature highlights that the need for social distancing reduced opportunities for face-to-face interaction and collaboration, resulting in a sense of professional isolation and detachment of the teaching staff from the students [5, 6]. This, in turn, had a negative impact on the quality of education and the learning experience of students [26–29]. Evidence also shows that the COVID-19 pandemic and its related measures brought a sense of dissatisfaction with work [12, 30]. Evidence is also available of widespread confusion about COVID-19-related information among school staff and disorientation, in general [31, 32]. A novel significant challenge that emerged was school staff’s pedagogical alienation, as a consequence of deontological distress. Participants in our study reported to be forced to navigate the implementation of new health measures while maintaining their pedagogical mandate, and recognized that these tasks were often at odds with each other. This suggests that participants had feelings of moral distress in their professional lives and experienced professional duty conflicts. Participants also reported to be witnessing the exacerbation of inequities. Remote learning widened the gap between students from disadvantaged backgrounds and their more privileged peers [33, 34]. Our study demonstrated that educators were forced to grapple with the challenge of providing equitable access to education in an altered setting, which requires a specific policy response (see Supplementary file, Recommendation 1).

Finally, we found that the COVID-19 pandemic and its associated measures significantly impacted the privacy of school staff. Participants reported feeling a substantial loss of privacy, with their private decisions becoming more visible and creating a moral tension. This highlights the need for policymakers to consider the privacy implications of any measures taken in response to a public health crisis (see Supplementary file, Recommendation 2).

Participants found creative solutions to continue performing their work optimally, often altering their moral standards in order to reach a given teaching objective. The literature shows that teachers demonstrated resilience and introduced creative solutions during the pandemic to adapt to the several changes in teaching and learning [35–39]. These solutions include changes to the format of lectures and instructional materials to facilitate self-paced learning, the creation of online discussion forums, group chats, and virtual classrooms to maintain student engagement and communication, increased support to students through one-on-one online meetings, encouragement of student collaboration and peer support through online group projects and activities, use of gamification and game-based learning strategies to increase student motivation and engagement, development of alternative assessment methods, and implementation of flexible schedules and asynchronous learning options to accommodate student’s individual needs and circumstances [40]. Our findings reveal that teachers fulfilled their moral and professional responsibilities as educators, adapting to the sudden health measures and the need to meet their teaching objectives. In other words, teachers may have made difficult decisions and sacrifices that challenged their personal and professional moral values or beliefs to ensure that students received the education they needed. This adaptation of moral standards is a unique aspect of the pandemic, and it highlights the exceptional circumstances that teachers had to navigate in fulfilling their professional obligations (see Supplementary file, Recommendation 3).

Participants noted that, while the COVID-19 pandemic and its measures presented numerous challenges to schools and their staff, there were some benefits to the measures implemented to contain the virus. For example, in line with other studies, our study noted that the pandemic raised awareness of public health and safety, with school staff learning about best practices for maintaining a safe and healthy environment for themselves and their students [40]. The pandemic highlighted the importance of communication and collaboration among school staff, with new platforms and tools being developed to facilitate communication and collaboration in a remote or hybrid work environment, and the essential role of educators in society, as they continued to work tirelessly to provide education and support to their students, often under difficult and challenging circumstances. Finally, the pandemic offered an opportunity to increased awareness of the importance of mental health, as schools recognized the need to provide mental health support and resources for their staff.

Participants in our study mentioned additional benefits that may be enhanced outside of the pandemic context in the long term, namely practicing gratitude, a better understanding of illness and death as part of life, and promotion of cultural diversity. Many school staff members reported that the pandemic helped them to appreciate the simple things in life and to practice gratitude. Practicing gratitude is a powerful tool that can help promote positive mental health and overall well-being, and which could be easily introduced in school programs. The pandemic brought illness and death to the forefront of our collective consciousness, and school staff are no exception. Participants in our study reported that the pandemic helped them to better understand illness and death as a natural part of life. This newfound understanding could lead to the promotion of greater empathy and compassion for students and colleagues who are dealing with illness or loss. Participants reported that the pandemic helped them become more aware of the importance of promoting cultural diversity and inclusivity in their classrooms. With the rise of anti-Asian hate crimes and discrimination during and after the pandemic, it is more important than ever to create a safe and inclusive learning environment for all students.

The individuals we interviewed were prompted to reflect on their experiences during the pandemic, which enabled them to make sense of the situation in terms of both opportunities and limitations. This process is crucial for learning, integrating experiences, and moving forward as enriched individuals (see Supplementary file, Recommendation 4).

### Limitations

One limitation of our study is that, despite initially securing collaboration with teacher’s unions during the study design phase, we faced some challenges during the recruitment stage. While this was a setback, we made every effort to recruit a diverse range of participants and ensure that our findings were still representative of the variety of the experiences of school staff during the pandemic. Further limitations are presented in the Supplementary file.

## Conclusions

Our study highlights the importance for education institutions and public health officials to account for the complexity of the professional and moral challenges which school staff confront in emergency situations such as a pandemic. It also stresses that it is crucial to listen to staff’s concerns, enacted strategies, and perceived benefits and take them into account when developing appropriate and effective public health and communication efforts. Understanding the unique challenges faced by different professional groups during a public health emergency is a crucial element in formulating effective public health policies that are widely adopted. Our study highlights the particular challenges faced by school staff, a distinct professional group, in adapting to the pandemic and its measures. Such understandings can help mitigate the negative effects of health emergencies on individual’s well-being and ensure the continued provision of critical services to communities and society.

## Supplementary Material

ckae196_Supplementary_Data

## Data Availability

Due to the sensitivity of the information provided by participants, data are only available upon request. Key points Our study documents the challenges posed by COVID-19 and its associated measures, explores diverse strategies implemented by school staff, and highlights reported benefits. Based on our insights, we propose recommendations emphasizing the importance of actively listening to staff concerns, understanding their implemented strategies, and acknowledging perceived benefits. These insights are crucial for informing robust public health policies and effective communication strategies tailored to current and future challenges in education and public health.
