# Supplementary material for: Impact of COVID-19 and related measures on the professional life of school staff based in Switzerland: challenges, strategies, and benefits
Source: Eur J Public Health. 2024 Dec 9;35(1):163–70. doi: 10.1093/eurpub/ckae196 (PMC11832152; doi:10.1093/eurpub/ckae196)
Supplement: ckae196_Supplementary_Data [file ckae196_supplementary_data.docx]

Supplementary file

Table S1. Methodological details

| Inclusion criteria | Staff included teaching staff (teachers, childcare workers, teaching assistants, speech therapists, paraprofessional educators), and other staff (office staff, bus drivers, school nurses and counsellors, school nutrition staff, coaches and athletic trainers, custodians, canteen staff, security staff, and cleaning staff). |
| --- | --- |
| Recruitment | This survey is part of a larger project on vaccine hesitancy among school staff conducted in Switzerland. At the end of the online survey, we included the question “Would you be willing to be interviewed to tell us more about your views?”. We included all the individuals who accepted to be interviewed and verified that the sample was diversified in terms of age, Canton of residence, working in rural and urban schools, working in private and public schools, and gender of the participants (f/m). |
| Data collection | All conversations were recorded and transcribed in the original language, without translation, to preserve nuances and context. The primary analyst was proficient in the language, allowing for accurate interpretation of the data without loss of meaning. Double coding was also implemented to enhance robustness, with discrepancies reviewed and resolved collaboratively. |
| Analysis | We employed thematic content analysis with a mainly inductive approach to analyze the data, enriched with abductive and deductive reasoning. In summary, the analysis initially followed an inductive approach: codes were generated directly from the data and then refined through iterative review. Once the coding structure was solidified, it was applied deductively to the data. The analysis proceeded as follows: in a first phase, we all analysed the same interviews, meeting weekly to discuss the analysis. In turn, one of the researchers then drew up an individual analytical sheet, which was then shared with the group and validated by the other researchers; as the individual analyses proceeded, one of the researchers took turns drawing up a cross-sectional analytical sheet, linking multiple interviews (again, the form was shared with the group and validated by the other researchers). The qualitative group thus worked closely together, reaching a consensus on the codes and their relationships. When signs of saturation appeared evident, the researchers drafted a final codebook, which was then used to deductively recode the interviews with a software in order to verify the analysis and ensure greater methodological rigor. At this stage, we conducted three additional interviews to confirm data saturation. Finally, we anonymized all data, removed any identifying information, replaced any names with pseudonyms, and translated the quotes into English. |
| Drafting of the results | After coding, we categorized participant responses into four main areas: professional challenges, strategies, benefits, and concerns. These categories were identified through an iterative coding process, where initial codes were refined and grouped into broader themes based on recurring patterns in the data. By doing so, we aimed to organize the results in a way that highlights participants’ experiences across these key dimensions. |

Table S2. Interview grid

| Themes and sub-themes | Questions |
| --- | --- |
| General experiences with the pandemic | |
| General experiences with the pandemic | We have now been in the midst of a global pandemic for more than 18 months. If you had to use three words to describe your general experience with the pandemic, what would they be? Tell us the meaning of the three words. |
| Hearing about access to the COVID-19 vaccines | Vaccines against COVID-19 are available from spring 2021. How did you receive this news? |
| Representation of COVID-19 vaccines | |
| Concerns about COVID-19 vaccines | When you think about COVID-19 vaccines, what are your concerns today? Can you name the three things that worry you most? |
| Positive aspects in relation to COVID-19 vaccines | When you think about the COVID-19 vaccines today, what are the aspects that make you feel better/relieved? Can you name the three aspects that comfort you the most? |
| Attitude towards federal policy | |
| Federal Policy | What do you think of the federal government’s policy on COVID-19 vaccines? What is your opinion on the green pass? What do you think about the use of the green pass in educational facilities? |
| vaccination decision and experience | |
| Vaccination decision | |
| Vaccination choice | Are you vaccinated? If not, do you intend to get vaccinated? |
| The decision-making process | In such a complex context, the decision to vaccinate or not to vaccinate is neither obvious nor easy. How did you come to your decision? What were the key moments in your decision? |
| Decision-making aids | What helped you make your decision?  What stood in your way/made it difficult for you to make a decision?  What do you think of the way the authorities “accompanied” you in the decision?  What do you think of the level of information provided? |
| Perceptions regarding the decision | How do you feel today about your decision regarding vaccination? Do you have second thoughts? Doubts? Regrets? Would you change anything if you could go back? |
| Vaccination experience | |
| Description of your vaccination | If you have already been vaccinated: Can you tell us about your experience with vaccination? How did it unfold? Can you mention three adjectives to describe this experience?  If you have not yet been vaccinated: Have you tried to get an appointment? If yes, how did it go? Why has the appointment not yet taken place? |
| Description of the vaccination of your acquaintances/family members | Do you know anyone who has been vaccinated? What has been their experience so far?  Did any of these experiences make you think? In what way? Do you think these experiences have influenced your current views on vaccination? |
| Role of the entourage | |
| Discussions, opinions and expectations | |
| Discussion of vaccination with the entourage | What is the climate like about the COVID-19 vaccination among your friends/family/at your workplace?  How often and in what ways do you talk about the COVID-19 vaccination with your friends/family/at work?  Is this a hot topic? How come? What’s being said about it? |
| Position of the entourage regarding vaccines | How do your family members feel about the COVID-19 vaccination?  What do your friends think about the COVID-19 vaccination?  What do your colleagues think about the COVID-19 vaccination?  What does your school’s leadership think about the COVID-19 vaccination?  What does your doctor think about the COVID-19 vaccination? |
| Reactions and expectations of the entourage | What do the people around you (family, friends, colleagues/superiors, caregivers, etc.) think about your decision to get vaccinated/not to get vaccinated?  How much did the people around you support/support you in your decision? In what way? Can you think of a situation where you felt really supported in your decision? What happened? Can you think of a situation where you did not feel supported? What happened?  In your opinion, what do your loved ones/acquaintances expect from you regarding the COVID-19 vaccination? Have you ever felt any pressure? How does this affect you? How does it make you feel? How do you manage these feelings? |
| Current way of life | |
| Living with/without the COVID-19 vaccination | |
| Impact of the decision on the way of life | How does your decision to get vaccinated/not to get vaccinated impact your daily life?  Have there been any changes because of your decision (e.g., in your relationships with others, your behaviors, the behaviors of others, etc.)?  Do you think there will be any changes in the future? Which ones? |
| Risk representation | How at risk do you feel about COVID-19 today? Why? |
| Risk management | If you are vaccinated, how are you protecting yourself and your students today?  If you plan to get vaccinated, how do you plan to protect yourselfyou’re your students in the meantime? And afterwards?  If you don’t plan to get vaccinated, how do you plan to protect yourself and your students? |
| Conclusion | |
| Vaccines in general | In general, how do you feel about vaccination?  Have you been vaccinated against the flu yet? |
| CAM | What is your relationship with alternative medicine? Do you use it? If so, how and why? |
| Future | How do you see the future? |
| Concerns | What worries you most? |
| Auspices | What do you hope for in general?  What do you hope for from the authorities, from the institutions, from your school? |
| Communication intervention preferences | |
| Persuasion | If you wanted to convince someone to get vaccinated, what would you say? What do you think are the main arguments in favour of vaccines?  If you wanted to convince someone not to get vaccinated, what would you say? What do you think are the main arguments against vaccines? |
| Preferred communication channels in view of a communication intervention on vaccines | If you wanted to receive information about the COVID-19 vaccination, what information would you want? Through which channels? How often would you like to receive this information? What form of communication would you prefer (e.g., video, audio, text)? Who would you like to pass this information on to you? |
| Greetings | |
| Comments | Is there anything important you would still like to share with us? |

Table S3. Recommendations

| Recommendation 1 | Policymakers need to recognize the unique challenges faced by school staff during such crises and provide them with the necessary support and resources to navigate them. This could include training and professional development opportunities to help school staff adapt to new teaching methods and technology, as well as access to mental health services and resources to help them cope with the stress and challenges of teaching during a pandemic. Efforts to limit the exacerbation of inequities in education could include investment in technology and infrastructure to ensure that all students have equitable access to education, as well as targeted interventions to support students from disadvantaged backgrounds or with special or higher educational needs. The implementation of hybrid teaching models that combine in-person and remote learning in and outside of pandemic times, could reduce distruption during emergencies and facilitate quality education, a lower sense of isolation and detachment, while also providing students with a more immersive and engaging learning experience. |
| --- | --- |
| Recommendation 2 | Policymakers should take steps to ensure that any measures implemented to address a crisis such as COVID-19 are designed with privacy protection in mind, and educators are adequately informed about how their privacy may be impacted. By doing so, they can ensure that privacy rights are not needlessly sacrificed in the pursuit of public health objectives. |
| Recommendation 3 | It may be advisable to reward school staff for embracing resilience and adhering to their mission in creative ways. In addition, it may be helpful to provide adequate support and resources to teaching staff, including training on ethical decision-making in challenging situations. This could minimize undue stress or burnout as they adapt to changing circumstances and make difficult decisions, and can help maintain the integrity of the educational system even in the face of significant challenges. |
| Recommendation 4 | It is important to provide individuals with the opportunity to reflect on their experiences, give them a name, and assign them meaning. Helping individuals integrate their experiences into their personal and professional trajectory is crucial, not only for educators but also for students. This approach should be considered in public health policies aimed at promoting overall well-being in the face of health crises. By enabling individuals to reflect on and make sense of their experiences, policies can foster resilience and facilitate growth in individuals and communities. |

Table S4. Limitations

| 1 | A limitation of our study is the potential for selection bias. We acknowledge that our sample may include a higher proportion of individuals who were politically dissatisfied with institutions and may have been motivated to participate in the study to express their opinions or raise their voices in a more militant way. While this may have influenced the overall views and experiences expressed by our participants, we tried to recruit a diverse range of participants and ensure that their voices were accurately represented. |
| --- | --- |
| 2 | A limitation is that we focused on only one stakeholder group. While such a focus is a strength in research, it does mean that we cannot compare how other important stakeholders (e.g., grocery store, pharmacy, or hospital staff) experienced the impact of COVID-19 and related measures in Switzerland on their professional life in terms of challenges, strategies, and benefits. |
